# Evaluation of ciprofloxacin and metronidazole encapsulated biomimetic nanomatrix gel on *Enterococcus faecalis* and *Treponema denticola*

**DOI:** 10.1186/s40824-015-0032-4

**Published:** 2015-06-01

**Authors:** Sagar N Kaushik, Jessica Scoffield, Adinarayana Andukuri, Grant C Alexander, Taneidra Walker, Seokgon Kim, Sung Chul Choi, Brigitta C Brott, Paul D Eleazer, Jin-Yong Lee, Hui Wu, Noel K Childers, Ho-Wook Jun, Jae-Hong Park, Kyounga Cheon

**Affiliations:** Department of Biomedical Engineering, University of Alabama at Birmingham, Birmingham, AL USA; Department of Pediatric Dentistry, University of Alabama at Birmingham, SDB 304B, 1720 2nd Ave S, Birmingham, AL 35294-0007 USA; Cardiovascular Division, School of Medicine, University of Alabama at Birmingham, Birmingham, AL USA; Department of Endodontics, University of Alabama at Birmingham, Birmingham, AL USA; Department of Pediatric Dentistry, School of Dentistry, Kyung Hee University, Seoul, Korea; Department of Maxillofacial Biomedical Engineering, Kyung Hee University, Seoul, Korea

**Keywords:** Ciprofloxacin, Metronidazole, *Enterococcus faecalis*, *Treponema denticola*, Injectable self-assembled biomimetic nanomatrix gel

## Abstract

**Background:**

A triple antibiotic mixture (ciprofloxacin; CF, metronidazole; MN, and minocycline; MC) has been used for dental root canal medicaments in pulp regeneration therapy. However, tooth discolorations, cervical root fractures, and inadequate pulp-dentin formation have been reported due to the triple antibiotic regimen. Therefore, an antibiotic encapsulated biomimetic nanomatrix gel was developed to minimize the clinical limitations and maximize a natural healing process in root canal infections. In this study, minimal bacterial concentrations (MBC) of the selected antibiotics (CF and MN) were tested in 14 representative endodontic bacterial species. Then MBC of each CF and MN were separately encapsulated within the injectable self-assembled biomimetic nanomatrix gel to evaluate antibacterial level on *Enterococcus faecalis* and *Treponema denticola*.

**Results:**

Antibiotic concentrations lower than 0.2 μg/mL of CF and MN demonstrated antibacterial activity on the 14 endodontic species. Furthermore, 6 different concentrations of CF and MN separately encapsulated with the injectable self-assembled biomimetic nanomatrix gel demonstrated antibacterial activity on *Enterococcus faecalis* and *Treponema denticola* at the lowest tested concentration of 0.0625 μg/mL.

**Conclusions:**

These results suggest that each CF and MN encapsulated within the injectable self-assembled biomimetic nanomatrix gel demonstrated antibacterial effects, which could be effective for the root canal disinfection while eliminating MC. In the long term, the antibiotic encapsulated injectable self-assembled biomimetic nanomatrix gel can provide a multifunctional antibiotic delivery method with potential root regeneration. Further studies are currently underway to evaluate the effects of combined CF and MN encapsulated within the injectable self-assembled biomimetic nanomatrix gel on clinical samples.

## Background

Dental pulp tissue exposed to mechanical trauma or cariogenic processes can result in root canals and/or periapical infections, which can be treated with endodontic procedure (root canal treatment) [1]. Infected teeth with immature root structure require a root end closure treatment (apexification) by Ca(OH)_2_ or mineral trioxide aggregate [2]. Yet the apexification method has been shown to result in poor pulp-dentin tissue formation. In order to regenerate pulp-dentin tissue in the infected immature root, a revascularization procedure has been applied using a triple antibiotics mixture, ciprofloxacin (CF), metronidazole (MN), and minocycline (MC), along with NaOCl irrigation and Ca(OH)_2_ [3-5]. However, the traditional revascularization treatment has been implicated in several adverse clinical outcomes including tooth discoloration, cervical fracture, and inadequate pulp-dentin tissue formation [6-8].

The regenerative tissue engineering concept has grown in the field of medicine. Its main contributors include pluripotent cells, signaling molecules, and scaffold system which is to capture characteristic properties of natural extracellular matrix (ECM) which plays a key role in tissue development [9-11]. The scaffolds can be synthesized by variety of recent nanotechnology including self-assembly, electrospinning and thermal inducing phase separation characterized by biocompatibility and biodegradability [10,12]. Among the nanotechnology, a biomimetic nanomatrix gel is formed by self-assembled peptide amphiphiles (PAs), which consist of a hydrophilic functional peptide sequence attached to a hydrophobic alkyl tail. Due to their amphiphilic characteristics and molecular shape, PAs can self-assemble into highly ordered ECM like nanostructures in optimal pH [13,14]. In addition, manipulative viscoelastic properties of PA structure allows for replication of the essential properties of the ECM environment [12,15-17]. Thus the biomimetic nanomatrix gel contains several functional units; scaffolding self-assembled nanofibers, injectable viscoelastic properties, encapsulation of cells or antibiotics at physiological conditions, releasing of antibiotics in a highly controlled manner, cell adhesive ligands, and enzyme-mediated degradable sites [16,18]. With these functional benefits, the biomimetic nanomatrix gel was considered to be applied to the regenerative endodontics; antibiotic molecules can be encapsulated within the self-assembled biomimetic nanomatrix gel to be released in controlled manner inside root canal, which can reduce the antibiotic concentrations compared to the triple antibiotic mixture; unique viscoelastic property of the nanomatrix gel enables direct injection into the infected root canal space; the ECM mimicking self-assembled PAs can promote interaction with surrounding pulp tissues. While the conventional triple mixture was mixed manually with nonfunctional and non-bioactive paste.

The goals of the successful endodontic regeneration therapy can be demonstrated by a lacking of post-treatment clinical symptoms and radiographic evidence of continued root development [3]. To achieve the goals, efficient root canal disinfection and adequate root dentin formation are proposed using antibiotics encapsulated injectable self-assembled biomimetic nanomatrix gel. In this study, minimal bactericidal concentrations (MBC) from the triple antibiotics, CF, MN excluding MC, were tested to determine bactericidal activity against 14 endodontic species. Augmentin (AM) was also tested as a potential alternative to MC to reduce the unfavorable tooth discoloration associated with the conventional triple mixture. Consequently, predetermined concentrations of the each antibiotic were encapsulated within the injectable biomimetic nanomatrix gel and evaluated its bactericidal activity on *Enterococcus faecalis* and *Treponema denticola. E. faecalis* and *T. denticola* were selected as the facultative and strict anaerobic species among the above 14 endodontic species.

## Methods

### Antibiotics and agar dilution preparation

The antibiotics, CF (Nelson Pharm Korea®, Seoul, Korea), MN (Cheil Jedang®, Seoul, Korea) and AM (Il-Sung Shin Yak®, Seoul, Korea), were provided by powder form and mixed with propylene glycol [5]. Each antibiotic was serially diluted in 2-fold from starting concentration 5 μg/mL and mixed with sterilized 1.5% agar supplemented with Brucella broth, Vitamin K1, Hemin solution, and supplemented with 5% defibrinated sheep blood in pH 7.2 as manufacture recommended (Becton, Dickinson and Company, Franklin Lakes, NJ, USA). Two mL of the mixed solution of the antibiotic and the media was distributed to the each well of 24 well-plates. Positive controls were prepared with 1 μL from 10-fold serial dilution of the bacteria culture broth without antibiotics. Antibiotics without bacterial species served as the negative control. Following preparation of the controls, the plates incubated overnight at room temperature.

### Bacterial species and culture

Fourteen bacteria species [19] which have been frequently observed from root canal infection were selected for the antibiotic susceptibility tests. The bacteria are *Aggregatibacter actinomycetemcomitans (Aa), Fusobacterium nucleatum (Fn), Porphyromonas endodontalis (Pe), Porphyromonas gingivalis (Pg), Prevotella nigresens (Pn), Prevotella intermedia (Pi), Tennerella forsythia (Tf), Treponema denticola (Td), Enterococcus faecalis (Ef), Lactobacillus casei (Lc), Streptococcus gordonii (Sg), Streptococcus mutans (Sm), Streptococcus sobrinus (Sso),* and *Streptococcus sanguinis (Ssa).* Each of the 14 bacterial species were incubated in anaerobic condition for 48 hours on Brucella blood agar plates; then several colonies were inoculated in suspension of 10 mL Brucella broth and incubated for 6 hrs anaerobically at 37°C as shown in the Table 1. The bacterial density from the suspensions was adjusted with sterile Brucella broth to equivalent by the 0.5 McFarland standard and the optical density (OD) value was adjusted to 0.1 [20]. From the adjusted broth culture of the 14 species, 1 μL was dropped in 5 different locations onto prepared the 1.5% agar in 24 well-plates within 15 minutes. Then the plates were incubated anaerobically at 37°C for 1–7 days depending on the species [21].

**Table 1:** **Common bacterial species found in endodontic root canals and culture condition**

**Bacterial species**	**Source**	**Media**		**Condition**
		**Liquid**	**Solid**	
*A. actinomycetemcomitans*	Y4	BHI broth	BHI + agar	37°C, 5% CO_2_
*F. nucleatum*	ATCC 23726	BHI broth	BHI + agar	37°C, Anaerobic
*P. endodontalis*	ATCC 35496	Pg broth	Blood agar	37°C, Anaerobic
*P. gingivalis*	2561	Pg broth	Blood agar	37°C, Anaerobic
*P. nigrescens*	ATCC 33563	Pg broth	Blood agar	37°C, Anaerobic
*P. intermedia*	ATCC 25611	Pg broth	Blood agar	37°C, Anaerobic
*T. forsythia*	ATCC 43037	PY borth	NAM medium	37°C, Anaerobic
*T. denticola*	ATCC 3521	TYGVS	TYGVS + agar	37°C, Anaerobic
*E. faecalis*	ATCC 4083	BHI broth	BHI + agar	37°C, Facultative
*L. casei*	HY 2782	MRS	MRS + agar	37°C, Facultative
*S. gordonii*	G9B	BHI broth	BHI + agar	37°C, Facultative
*S. mutans*	GS5	BHI broth	BHI + agar	37°C, Facultative
*S. sobrinus*	6715	BHI broth	BHI + agar	37°C, Facultative
*S. sanguinis*	clinical isolates	BHI broth	BHI + agar	37°C, Facultative

BHI: Brain-heart infusion, Pg: Porphyromonas gingivalis , TYGVS: tryptone-yeast extract-gelatin-volatile fatty acids-serum, MRS: deMan, Rogosa and Sharpe, NAM: N-acetylmuramic acid.

The data was initially measured as Minimal Inhibitory Concentration (MIC); however, the MIC can be considered as MBC when it shows 99.9% of bacterial colony reduction (bactericidal effects). For bactericidal drugs, the MBC is usually the same as and generally not more than 4-fold higher than the MIC [22]. Therefore, MBCs were determined by counting colony forming units (CFUs) at 99.9% of bactericidal effect of each of the 14 species based on the anaerobe antimicrobial susceptibility testing protocol [23].

### Synthesis of peptide amphiphiles

The peptide amphiphiles (PAs) were synthesized using standard Fmoc-chemistry on an Advanced Chemtech Apex 396 peptide synthesizer (AAPPTec, Louisville, KY, USA), as described before [14]. The three different peptides, Tyr-Ile-Gly-Ser-Arg, (YIGSR, cell-adhesive ligand), Lysine (KKKKK, NO donor), and Short (enhance gelation) were synthesized to be 13 amino acids long and contain the MMP-2 sensitive sequence (GTAGLIGQ) [14]. Following the synthesis, the peptides were alkylated through linkage to a 16 carbonpalmityl chain resulting in PAs: PAs C16-GTAGLIGQ-YIGSR (PA-YIGSR), C16-GTAGLIGQ-KKKKK (PA-KKKKK), and C16-GTAGLIGQ-S (PA-Short). PA-YIGSR and PA-KKKKK were dissolved in deionized water to prepare 1 wt % stock solutions. The pH of these solutions was adjusted to 7.4 using a 1 M sodium hydroxide solution. PA-Short was dissolved in deionized water to make a 2 wt % stock solution. The pH of this solution was also adjusted to 7.4 in the same manner. PA-YIGSR and PA-KKKKK were then mixed in a ratio 9 PA-YIGSR: 1 PA-KKKKK to form PA-YK. The 9:1 ratio for PA-YK was selected due to its optimized performance in gelation as cell adhesion is significantly improved with increasing PA-YIGSR concentration as previously demonstrated [24]. Then 25 μL of PA-YK and 25 μL of the PA-Short solutions were placed in 12-well silicon inserts, flexiPerm® (Sigma Aldrich, St. Louis, MO, USA), attached to a glass cover slide.

### Encapsulation of ciprofloxacin (CF) and metronidazole (MN) in the biomimetic nanomatrix gel

After the screening of the 14 endodontic species, the antibiotics CF (GenHunter, Nashville, TN, USA) and MN (Sigma-Aldrich, St. Louis, MO, USA) were purchased and separately prepared as 5 μg/mL stock solutions. Then the antibiotic stock solutions were serially diluted to concentrations of 0, 0.0625, 0.125, 0.25, 0.5 and 1 μg/mL in 15 mL of DI water at pH 7.4. Ten μL of an aqueous solution of 0.1 M CaCl_2_ and 5 μL of the prepared antibiotic solution were added to a cylindrical-shaped silicone mold flexiPerm® to induce self-assembled antibiotic encapsulated within the biomimetic nanomatrix gel. The cylindrical-shaped gel was preferred for the intra-canal injectable biomimetic nanomatrix gel application.

### Bacterial culture for *E. faecalis* and *T. denticola*

**Figure 1:**
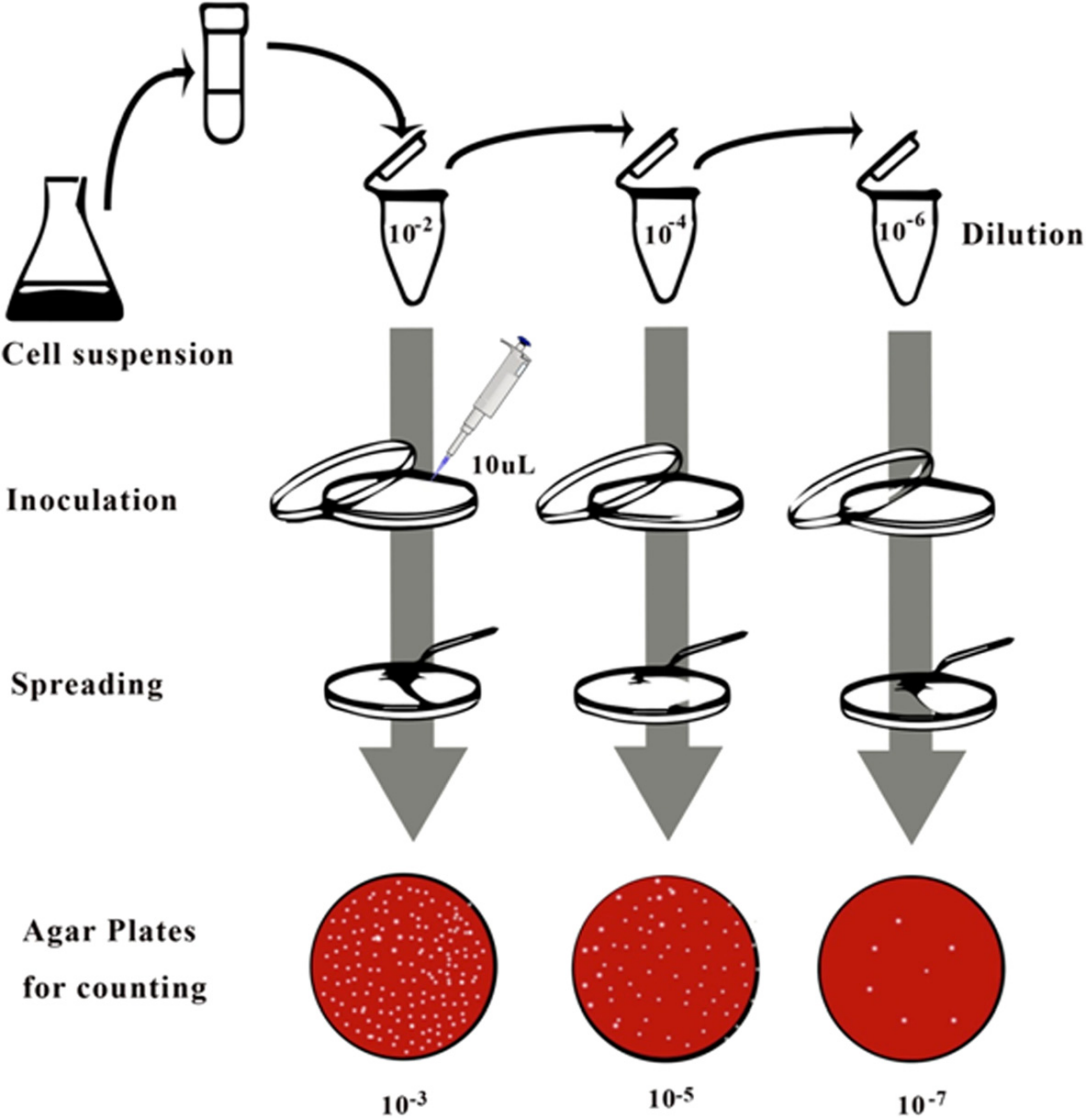
Bacterial morphology and colony forming units (CFUs)/mL. Bacterial morphology and CFUs were verified with serial dilutions followed by sheep blood agar culture. Optical Density (600 nm) measured 10^−4^ dilution tube.

*E. faecalis* was cultured in Todd-Hewitt broth (THB) and incubated aerobically with 37°C with 5% CO_2_ for 24 hours. Similarly, *T. denticola* was cultured in New Oral Spirochete media (NOS) and grown in a Coy anaerobic chamber at 37°C for 24 hours. Overnight bacterial cultures were sub-cultured by diluting 1/100 in THB or NOS until the bacteria reached mid-log phase. To determine CFUs, *E. faecalis* was serially diluted and plated on sheep blood agar (BD Falcon, Bedford, MA, USA) at 37°C for 24 hours. The bacterial densities from the broth suspensions were determined by the 0.5 McFarland standard with optical density (OD_600_); *E. faecalis* (6.2 × 10^6^ CFU/mL) and *T. denticola* (2.0 × 10^9^ CFU/mL) were used throughout the experiment [20,25]. As seen in Figure 1, the CFUs were counted by serially diluted cell suspension: 10^−2^, 10^−4^ and 10^−6^. Ten μL of each serially diluted bacterial suspension was pipetted into an Anaerobe Blood Agar Plate (BD Falcon, San Jose, CA) and spread for 24 hr incubation. The plates were visually inspected to determine the colony morphology and counted for CFUs.

### Evaluation of antibacterial effects of the antibiotic encapsulated injectable biomimetic nanomatrix gel

**Figure 2:**
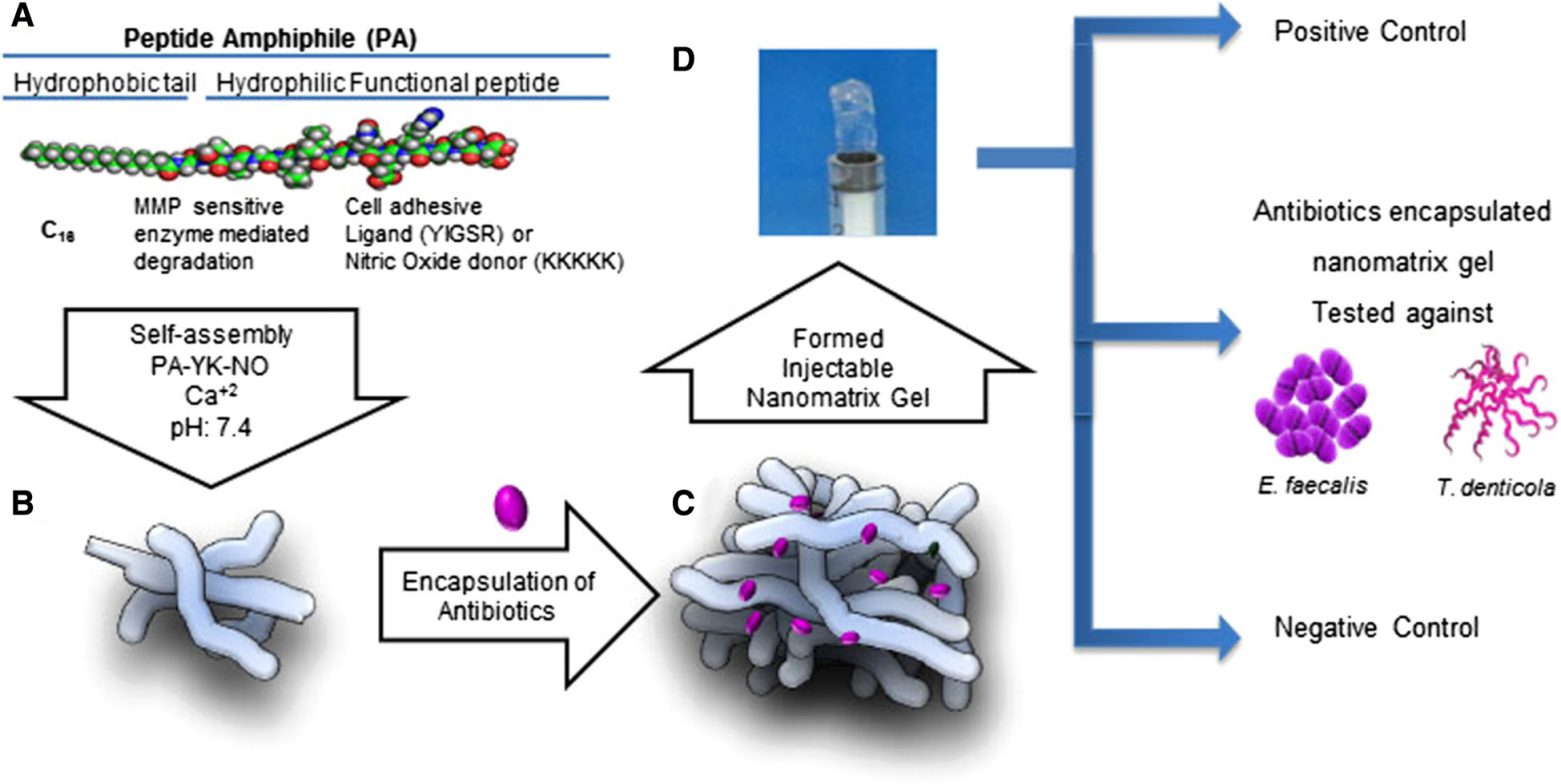
General scheme of the experimental design. Antibiotic encapsulated biomimetic nanomatrix gel system was evaluated against the aerobic bacteria, *E. faecalis* and anaerobic bacteria, *T. denticola* with three treatment conditions: CF, MN, and no antibiotic*.* **A**. Synthesis of peptide amphiphiles (PAs), **B**. Self-assembly of PAs, **C**. Encapsulation of antibiotics, **D**. Formation of injectable nanomatrix gel and experiment design by with three options: Positive control (antibiotic without biomimetic nanomatrix gel), Antibiotic (ciprofloxacin: CF and metronidazole: MN) encapsulated biomimetic nanomatrix gel, Negative control (bacteria only).

The prepared cylindrical nanomatrix gels were removed from the flexiPerm® and placed in the center of each 24well culture plate. One mL of the 24 hour-cultured THB bacteria was evenly distributed in each of the wells and incubated in aerobic or anaerobic condition, depending on the bacteria, at 37°C for 24 hours. Positive controls (antibiotic without nanomatrix gel) and negative controls (bacteria only) were also included as shown in Figure 2. The experiments were repeated 4 times for calculation of the mean values. ODs were used to measure bacterial loads at the variable antibiotic concentrations (0 μg/mL, 0.0625 μg/mL, 0.125 μg/mL, 0.25 μg/mL, 0.5 μg/mL and 1 μg/mL) using a spectrophotometer (Beckman Coulter DU800, Brea, CA, USA). To determine the bactericidal activity after 24 hour culture, ODs were measured by collection of 500 μL from the each wells of the 24 well bacteria culture plates and measured using a disposable cuvette at a wavelength of 600 nm [26,27] using a spectrophotometer.

## Results

### Minimal bactericidal concentration (MBC) of 3 antibiotics

Table 2 summarizes MBCs of the 3 antibiotics (CF, MN, and AM), respectively for the 14 species cultures. The MBCs for both CF and MN were 0.1 μg/mL in 6 species (*Aa, Fn, Pe, Pg, Pi, and Tf*) and 0.2 μg/mL in 5 species (*Ef, Sg, Sm, Sso, and Ssa*). Meanwhile the MBC for AM was required 2 to 5-fold higher concentrations to achieve similar effect to CF and MN.

**Table 2:** **Minimal bactericidal concentration (MBC) of 3 antibiotics measured for 14 bacterial species**

**Species**	**MBC (μg/mL)**		**Species**	**MBC (μg/mL)**	
*A. actinomycetemcomitans*	CF	0.1	*T. denticola*	CF	0.1
	MN	0.1		MN	0.25
	AM	0.5		AM	0.5
*F. nucleatum*	CF	0.1	*E. faecalis*	CF	0.2
	MN	0.1		MN	0.2
	AM	0.5		AM	1
*P. endodontalis*	CF	0.1	*L. casei*	CF	1
	MN	0.1		MN	0.5
	AM	0.5		AM	2
*P. gingivalis*	CF	0.1	*S. gordonii*	CF	0.2
	MN	0.1		MN	0.2
	AM	0.5		AM	1
*P. nigresens*	CF	0.25	*S. mutans*	CF	0.2
	MN	0.25		MN	0.2
	AM	0.5		AM	1
*P.intermedia*	CF	0.1	*S. sobrinus*	CF	0.2
	MN	0.1		MN	0.2
	AM	0.5		AM	1
*T. forsythia*	CF	0.1	*S. sanguinis*	CF	0.2
	MN	0.1		MN	0.2
	AM	0.5		AM	1

CF: ciprofloxacin, MN: metronidazole, AM: Augmentin, MBC: minimal bactericidal concentration.

### The effect of ciprofloxacin (CF) and metronidazole (MN) against facultative anaerobic *E. faecalis*

**Figure 3:**
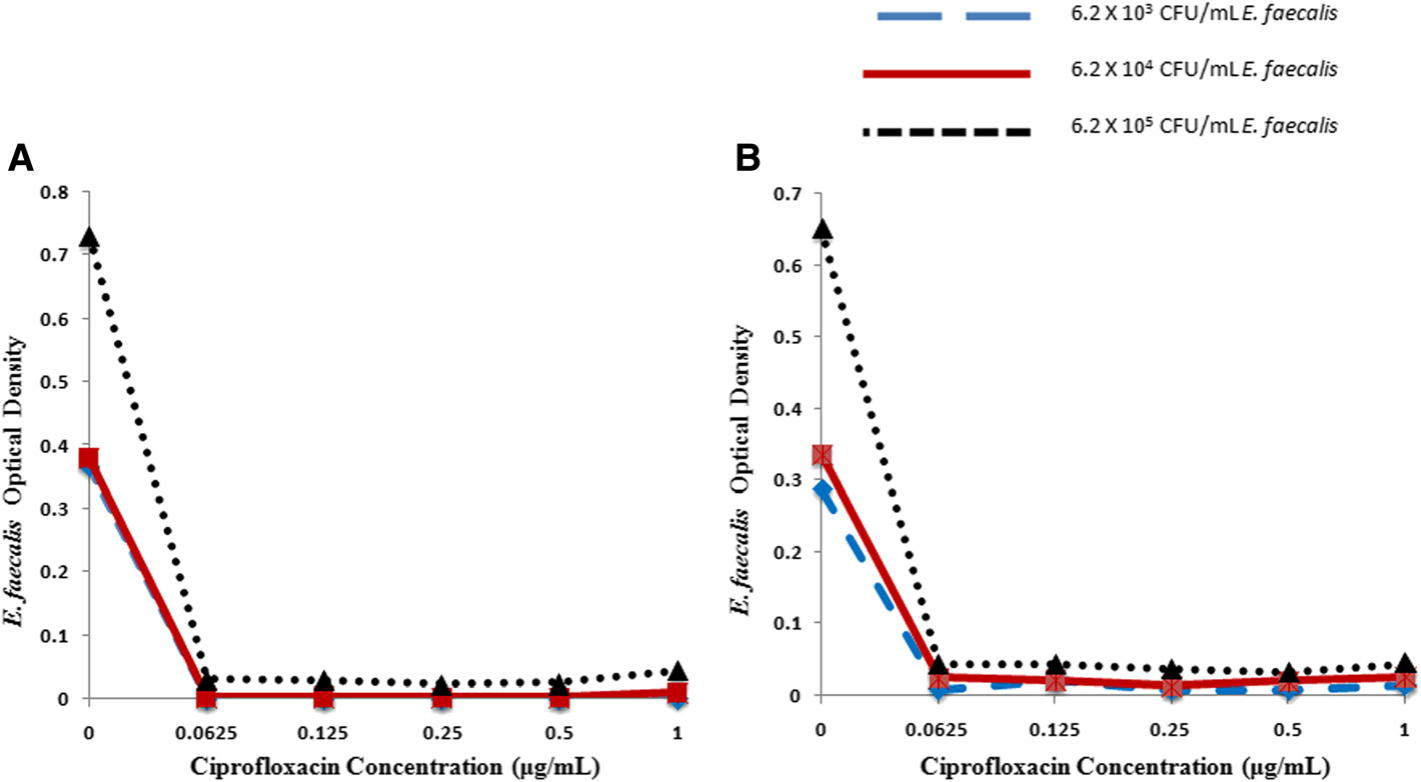
The effect of ciprofloxacin (CF) against *E. faecalis.* Antibacterial effect of CF was measured in varying concentrations of 0 μg/mL, 0.0625 μg/mL, 0.125 μg/mL, 0.25 μg/mL, 0.5 μg/mL and 1 μg/mL against *E. faecalis* in concentrations of 6.2 × 10^3^ CFU/mL, 6.2 × 10^4^ CFU/mL and 6.2 × 10^5^ CFU/mL*.* **A**. Without nanomatrix gel, **B**. With antibiotic encapsulated in nanomatrix gel.

CF was shown to have dose dependent antibacterial effect against the three different *E. faecalis* densities at the lowest concentration of 0.0625 μg/mL (Figure 3A). Figure 3B illustrated that the CF encapsulated biomimetic nanomatrix gel was tested in the three different *E. faecalis* densities and had similar antibacterial effects as CF without biomimetic nanomatrix gel.

**Figure 4:**
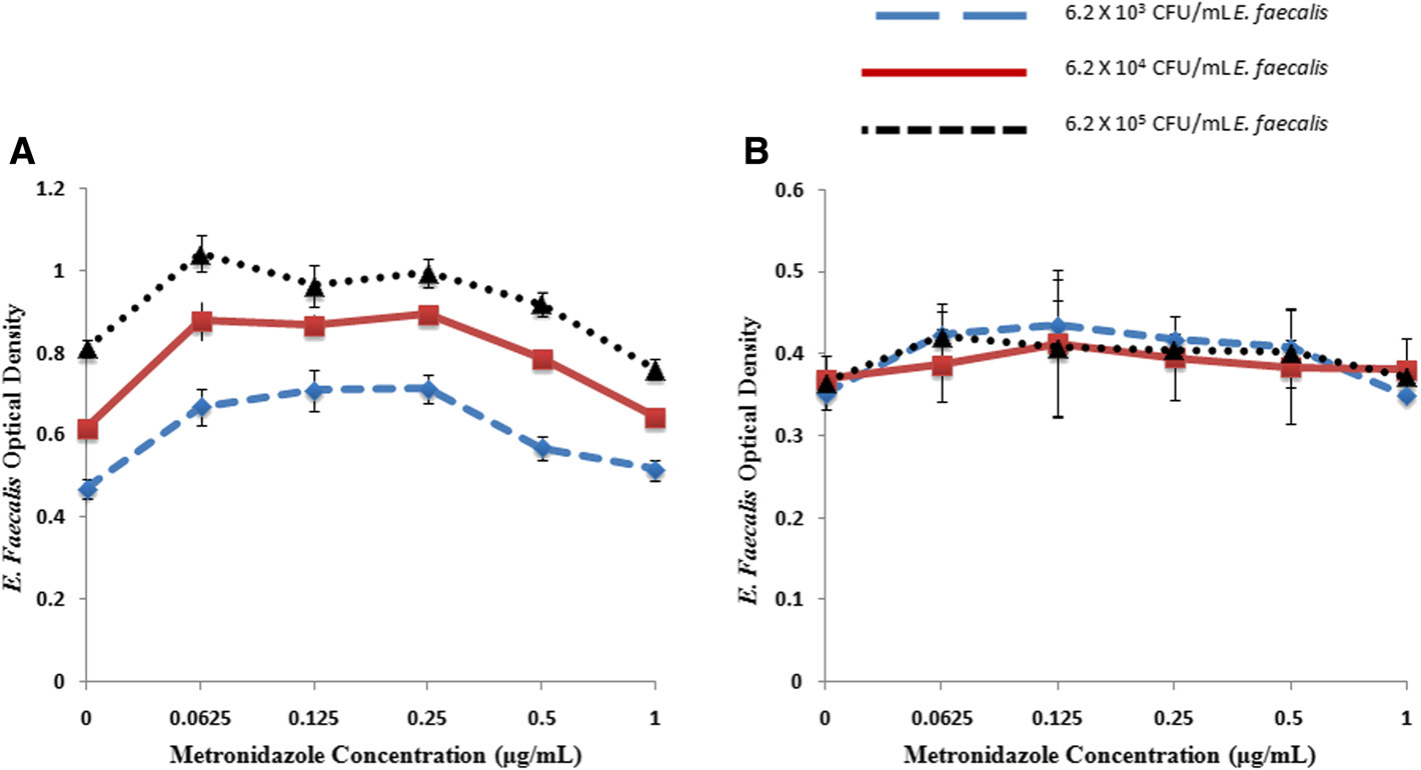
The effect of metronidazole (MN) against *E. faecalis.* Antibacterial effect of MN was measured in varying concentrations of 0 μg/mL, 0.0625 μg/mL, 0.125 μg/mL, 0.25 μg/mL, 0.5 μg/mL and 1 μg/mL on *E. faecalis* in concentrations of 6.2 × 10^3^ CFU/mL, 6.2 × 10^4^ CFU/mL and 6.2 × 10^5^ CFU/mL. **A**. Without nanomatrix gel, **B**. With antibiotic encapsulated in nanomatrix gel.

The effect of the antibiotic MN was studied on the same densities of *E. faecalis* as the CF experiments. In Figure 4, interestingly the antibiotic MN was not as effective on *E. faecalis* as CF was. The results of the MN encapsulated with nanomatrix gel in same bacterial densities also did not show antibacterial effect (Figure 4B).

### The effect of ciprofloxacin and metronidazole against anaerobic *T. denticola*

**Figure 5:**
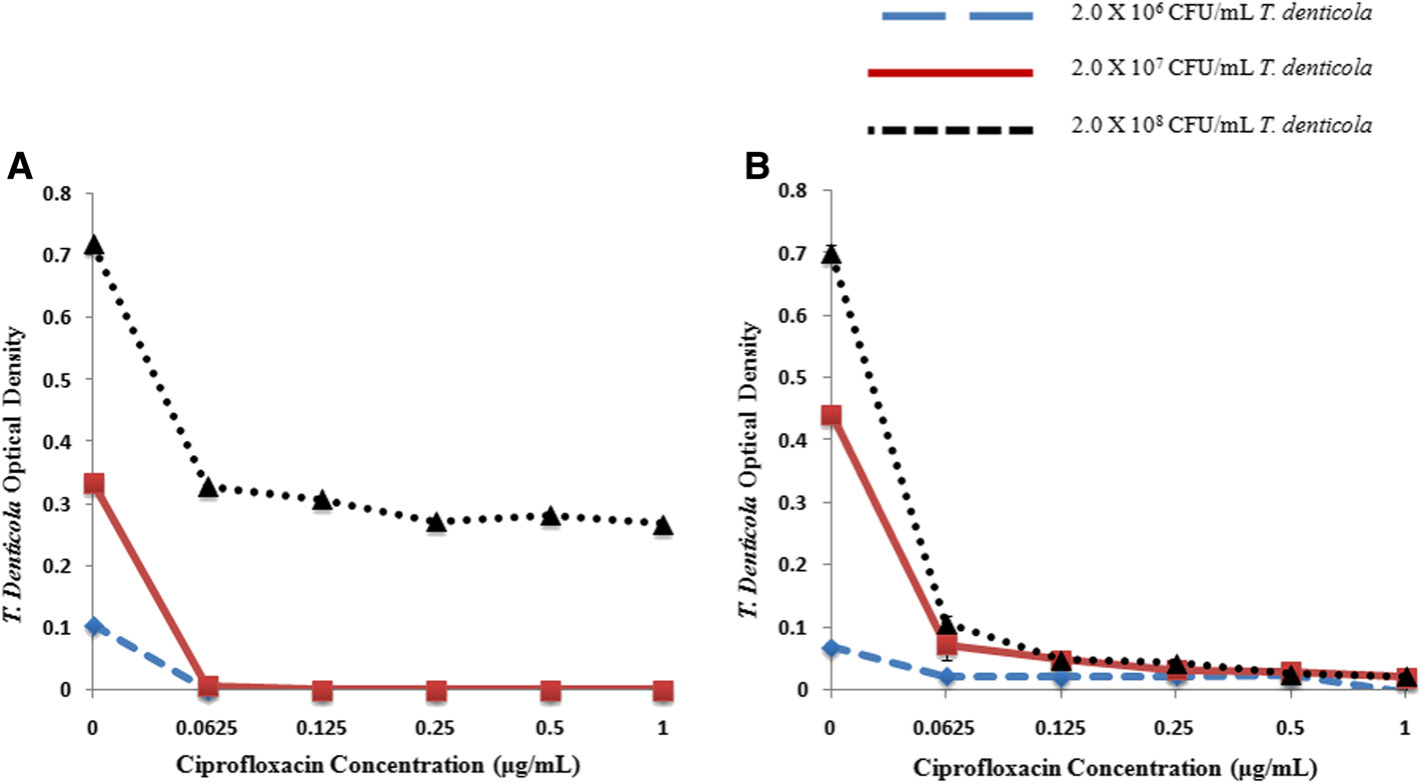
The Effect of ciprofloxacin (CF) against *T. denticola.* Antibacterial effect of CF was measured in varying concentrations of 0 μg/mL, 0.0625 μg/mL, 0.125 μg/mL, 0.25 μg/mL, 0.5 μg/mL and 1 μg/mL on *T. denticola* in concentrations of 2.0 × 10^6^ CFU/mL, 2.0 × 10^7^ CFU/mL and 2.0 × 10^8^ CFU/mL. **A**. Without nanomatrix gel, **B**. With antibiotic encapsulated in nanomatrix gel.

As seen in Figure 5A, the antibiotic CF against *T. denticola* displayed complete bactericidal effect in the different densities (2.0 × 10^6^ CFU/mL and 2.0 × 10^7^ CFU/mL) at the concentration of 0.0625 μg/mL without biomimetic nanomatrix gel*.* Similarly, the CF encapsulated with biomimetic nanomatrix gel (Figure 5B) showed that the antibacterial activity is effective at 0.0625 μg/mL concentration to all bacterial densities including 2.0 × 10^8^ CFU/mL.

**Figure 6:**
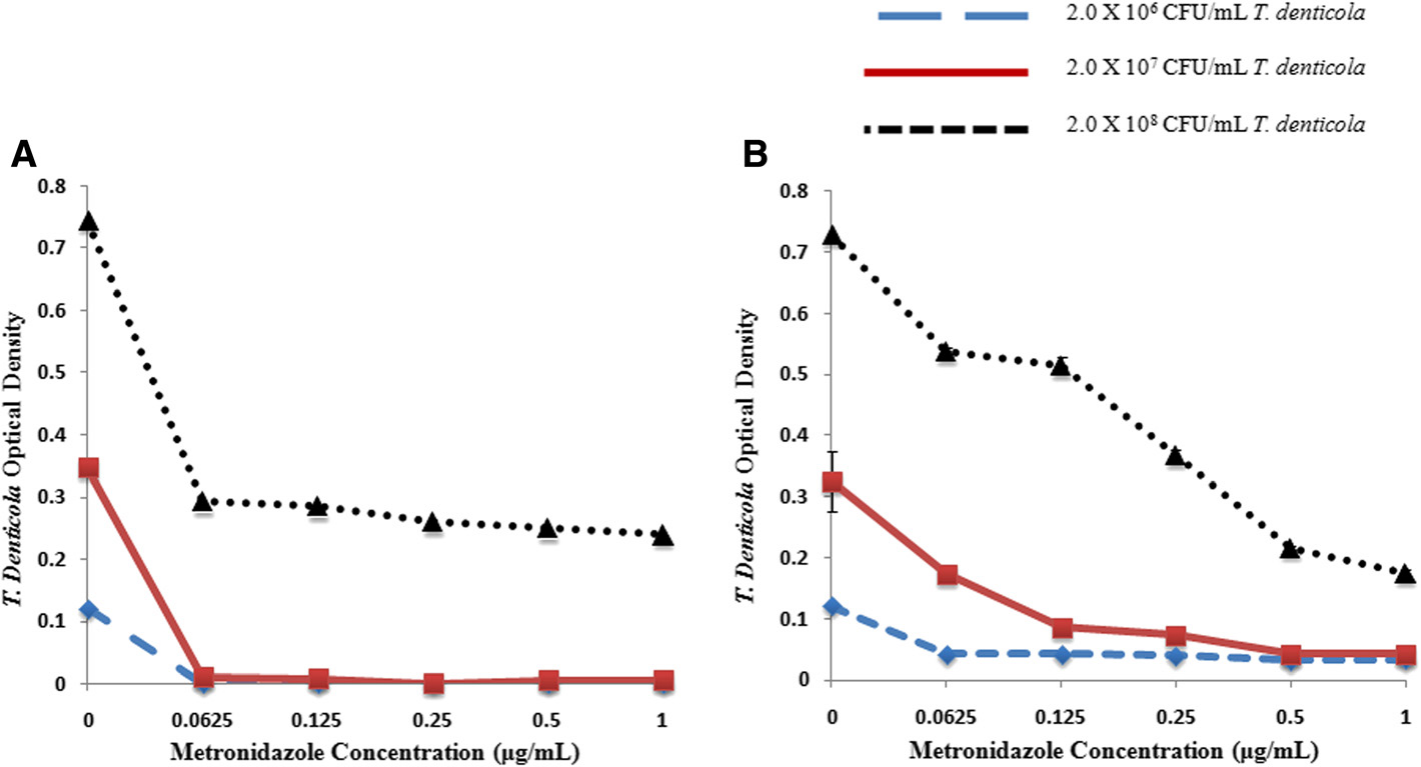
The Effect of metronidazole (MN) against *T. denticola.* Antibacterial effect of MN was measured in varying concentrations of 0 μg/mL, 0.0625 μg/mL, 0.125 μg/mL, 0.25 μg/mL, 0.5 μg/mL and 1 μg/mL on *T. denticola* in concentrations of 2.0 × 10^6^ CFU/mL, 2.0 × 10^7^ CFU/mL and 2.0 × 10^8^ CFU/mL. **A**. Without nanomatrix gel, **B**. With antibiotic encapsulated in nanomatrix gel.

The antibacterial effect of MN was seen on the bacterium *T. denticola* with varying densities as shown in Figure 6. Bactericidal effects were shown at 0.0625 μg/mL of MN without encapsulation in an injectable self-assembled biomimetic nanomatrix gel (Figure 6A) and with encapsulation in an injectable self-assembled biomimetic nanomatrix gel (Figure 6B).

## Discussion

### Antimicrobial susceptibility test

Table 2 displays the MBCs of the three antibiotics (CF, MN, and AM). CF interferes with DNA function in bacteria such as aerobic and anaerobic gram-positive bacteria and several mycobacterium species [28]. MN interferes with bacterial DNA of gram-negative anaerobic bacilli [29]. AM, a combination of amoxicillin and clavulanate potassium, has shown great efficacy in dental infection due to its broad spectrum and few adverse effects [30,31]. AM was tested instead of MC which is the most effective component; although its adverse effect of tooth discoloration is detested in clinical applications [6]. When the MBCs were converted to a ratio, it was observed as 1:1:5 (CF: MN: AM) respectively in 11 species (*Aa, Fn, Pe, Pg, Pi, Tf, Ef, Sg, Sm, Sso, and Ssa*), which ratio reflect that AM required 2 to 5-fold higher concentrations overall to achieve similar bactericidal effects compared to CF and MN. Therefore, AM was removed from the nanomatrix encapsulation study due to concern of increased concentration dosage affecting the patients’ health. CF and MN have successfully been used in intra-canal medicaments without incorporating a third component, MC, and are commonly referred to as double antibiotic paste [32]. Furthermore, our results indicate that CF and MN can be effective with a 1:1 ratio concentration than the triple antibiotic ratio (1:3) and agreed to the previous study [32].

Recent *in vitro* studies regarding the cytotoxicity of the single antibiotic have shown that the concentrations of 0.024 μg/mL maintained dental pulp cell viability for during 7 days [33] and more than 1 mg/mL can be a harmful effect on dental pulp stem cell [34]. This supports our results which demonstrated between 0.0625 μg/mL and 1 μg/mL for a single antibiotic bactericidal effect. Although our data is preliminary, it suggests potential benefits to patients by reducing unfavorable antibiotic complications. Further investigations will be required to confirm our findings in clinical samples, which may include a complex of endodontic bacteria.

Table 2 results also show that the latter 5 species (*Ef, Sg, Sm, Sso, and Ssa*) among the 11 species with a 1:1:5 MBC ratio were all facultative anaerobes and required an antibiotic concentration that was double that of the other 6 species (*Aa, Fn, Pe, Pg, Pi and Tf*). This may be due to the survival capability of the facultative anaerobes under the anaerobic environment. Particularly, *E. faecalis* is known as normal commensal flora in the human digestive system and often causes nosocomial infections and has been shown to display antibiotic resistance. It is also known as one of the main pathogens causing recurrent endodontic infections [35].

### Selection of *E. faecalis* and *T. denticola*

Infective dental root canal consisted of a variety of bacteria; among the endodontic microbiota, *E. faecalis* and *T. denticola* were selected as an initial microorganism to be tested for antibiotic encapsulated self-assembled biomimetic nanomatrix gel. *E. faecalis,* a gram-positive aerobic cocci and *T. denticola*, a gram-negative anaerobic fusiform rod, are often found in endodontic infections and commonly used to evaluate endodontic disinfectants [36,37]. As a pilot study, these two species *in vitro* studies demonstrated the optimal bactericidal concentrations of antibiotic encapsulated injectable self-assembled biomimetic nanomatrix gel delivery system.

### Antibiotic encapsulation within the injectable self-assembled biomimetic nanomatrix gel

As observed in Figures 3 and 5, when CF was encapsulated in the injectable self-assembled biomimetic nanomatrix gel (Figures 3B and 5B), CF was successfully released from the injectable self-assembled biomimetic nanomatrix gel and demonstrated similar bactericidal effect as antibiotic itself. This indicated that the injectable self-assembled biomimetic nanomatrix gel did not restrict the release and function of CF and also possibly suggests a sustained release of antibiotic from the injectable self-assembled biomimetic nanomatrix gel, which is effective against residual root canal bacteria. The results of Figure 5B also indicated that a sustained release of antibiotic via self-assembled injectable biomimetic nanomatrix gel promote a greater bactericidal effect than one time release of the antibiotics as seen in Figure 5A. Interestingly, the results of MN against *E. faecalis* (Figure 4A and B) demonstrated no bactericidal effects on both with and without injectable self-assembled biomimetic nanomatrix gel; this reflected that MN may not be an effective antibiotic choice for facultative anaerobic bacteria [38]. “In Figure 4B, the condition of the culture media and *E. faecalis* together may interact with the nanomatrix gel and affect the OD values of the experiment and this culture condition will be investigated further in the future experiment.” However, MN may be the effective bactericidal agent in a complex of bacteria from the root canal infection, which represents most of anaerobic bacteria. When MN encapsulated injectable self-assembled biomimetic nanomatrix gel was tested against *T. denticola* (Figure 6A and B); bactericidal effect has shown successfully in the two lower *T. denticola* densities (2.0 × 10^6^ CFU/mL and 2.0 × 10^7^ CFU/mL). In the density of 2.0 × 10^8^ CFU/mL, *T. denticola* did not portray complete bactericidal activity and this may be relatively low MN concentrations compare to the bacterial load.

The key concept of our study is that the self-assembled PAs can be mixed with antibiotics as a direct injectable material into the infected root canal, which will provide aseptic root canal and further provide a semi-natural ECM environment. Although cellular viability of the biomimetic nanomatrix gel for the dental pulp tissue has not been studied yet; other studies have successfully shown excellent cellular biocompatibility, high level of cell confluence, and cell migration in cardiac tissues [16,39]. Our current study will be an important fundamental step to assess not only the effectiveness of MN and CF on infectious root canal bacteria, but also the potential development of the direct injectable self-assembled biomimetic nanomatrix gel to treat endodontic infections including necrotic immature teeth. As a next step, 1:1 combination of CF: MN encapsulated within injectable self-assembled biomimetic nanomatrix gel will be tested for a complex of endodontic root canal bacteria from patient samples *in vitro* and *in vivo* experiments. In addition, the characteristics of antibiotic release from the biomimetic nanomatrix gels will be studied to explain the duration and intensity of the antibiotic effects.

## Conclusions

Concentrations (0.0625-0.25 μg/mL) of each CF and MN encapsulated within the injectable biomimetic nanomatrix gel successfully demonstrated antibacterial activity on *E. faecalis* and *T. denticola.* The proposed antibiotic encapsulated injectable biomimetic nanomatrix gel suggested an effective disinfecting and preconditioning root canal treatment with numerous potential benefits as innovative endodontic materials.

## Abbreviations

CF: Ciprofloxacin
MN: Metronidazole
MC: Minocycline
ECM: Extracellular matrix
PAs: Peptide amphiphiles
MBC: Minimal bactericidal concentration
OD: Optical density
MIC: Minimal inhibitory concentration
CFUs: Colony forming units
THB: Todd-Hewitt broth
NOS: New Oral Spirochete media
